# Isolation of a Novel *Streptomyces* Species from the Tuha Basin and Genomic Insights into Its Environmental Adaptability

**DOI:** 10.3390/microorganisms13102238

**Published:** 2025-09-24

**Authors:** Xiaomin Niu, Yujie Wu, Xue Yu, Shiyu Wu, Gaosen Zhang, Guangxiu Liu, Tuo Chen, Wei Zhang

**Affiliations:** 1 State Key Laboratory of Ecological Safety and Sustainable Development in Arid Lands, Northwest Institute of Eco-Environment and Resources, Chinese Academy of Sciences, Lanzhou 730000, China; niuxiaomin22@mails.ucas.ac.cn (X.N.);; 2 Key Laboratory of Extreme Environmental Microbial Resources and Engineering of Gansu Province, Northwest Institute of Eco-Environment and Resources, Chinese Academy of Sciences, Lanzhou 730000, China; 3 University of Chinese Academy of Sciences, Beijing 100049, China; 4 State Key Laboratory of Cryospheric Sciences and Frozen Soil Engineering, Northwest Institute of Eco-Environment and Resources, Chinese Academy of Sciences, Lanzhou 730000, China

**Keywords:** *Streptomyces*, polyphasic taxonomy, Tuha Basin, genomics, secondary metabolite potential

## Abstract

Mining novel *Streptomyces* species from extreme environments provides a valuable strategy for the discovery of new antibiotics. Here, we report a strain of *Streptomyces* sp. HMX87^T^, which exhibits antimicrobial activity and was isolated from desert soil collected in the Tuha Basin, China. Molecular taxonomic analysis revealed that the 16S rRNA gene sequence of strain HMX87^T^ shares the highest similarity with those of *Streptomyces bellus* CGMCC 4.1376^T^ (98.5%) and *Streptomyces coerulescens* DSM 40146^T^ (98.43%). In phylogenetic trees, it formed a distinct branch. The average nucleotide identity (ANI) and digital DNA–DNA hybridization (dDDH) values between strain HMX87^T^ and the above two type strains were below the thresholds of 95% and 70%, respectively, confirming that strain HMX87^T^ represents a novel species within the genus *Streptomyces*, for which the name *Streptomyces hamibioticus* sp. nov. is proposed. Physiologically, the strain HMX87^T^ grew at temperatures ranging from 25 to 37 °C, tolerated pH values from 5 to 12, and survived in NaCl concentrations of 0% to 8% (*w*/*v*). Chemotaxonomic characterization indicated the presence of LL-diaminopimelic acid (LL-DAP) in the cell wall, ribose and galactose as whole-cell hydrolysate sugars, MK-9(H8) (66.3%) as the predominant menaquinone, and iso-C_16:0_ (25.94%) and anteiso-C_15:0_ (16.98%) as the major fatty acids characteristics that clearly distinguish it from its closest relatives. Whole-genome sequencing of strain HMX87^T^ revealed an abundance of genes associated with high-temperature tolerance, salt-alkali resistance, and antimicrobial activity. The genomic features and secondary metabolic potential reflect its adaptation to extreme environmental conditions, including high temperature, salinity, alkalinity, strong ultraviolet radiation, and oligotrophic nutrients. The strain HMX87^T^ has been deposited in the Czech Collection of Microorganisms (CCM 9454^T^) and the Guangdong Microbial Culture Collection Center (GDMCC 4.391^T^). The 16S rRNA gene and whole-genome sequences have been submitted to GenBank under accession numbers PQ182592 and PRJNA1206124, respectively.

## 1. Introduction

Deserts, characterized by extreme environmental conditions such as high temperature, aridity, and saline-alkaline stress, along with associated pressures including intense inorganic oxidation, scarcity of organic carbon, dramatic temperature fluctuations, and strong solar radiation, exhibit severely limited biodiversity [1]. These conditions support only a limited number of highly adapted and specialized life forms [2]. Such environments shape unique soil microbial communities, which play crucial roles in biogeochemical cycles such as carbon and nitrogen cycling [3,4,5]. Studies on microbial diversity in the deserts of northern China have revealed that Actinobacteria constitute the dominant bacterial phylum in desert soils [6]. They are not only involved in the decomposition of organic matter and complex polymers—thereby facilitating global carbon cycling—but also contribute to enhanced plant productivity and stress resistance [7,8]. According to current estimates, microbial natural products (NPs) are encoded by over 200,000 genomic entries, harboring a rich diversity of biosynthetic gene clusters (BGCs) [9]. In a single actinobacterial genome—considered among the most promising sources for NPs research—up to 30 BGCs may be present, the majority of which have not yet been characterized. These gene clusters enable actinobacteria to produce a wide array of bioactive metabolites, including antibiotics, anticancer agents, immunosuppressants, enzymes, enzyme inhibitors, and other therapeutic or bioactive compounds [10,11,12,13,14].

According to statistics, between 2000 and 2021, a total of 129 new species were reported across 35 deserts worldwide [15]. As a key group within Actinobacteria, the genus *Streptomyces* was one of the most prolific sources of novel species [15]. Among the over 1000 microbially derived bioactive natural products identified to date, approximately two-thirds of antimicrobial compounds originate from secondary metabolites produced by *Streptomycetes* [16]. This highlights the indispensable role of *Streptomyces* in the discovery and development of novel antibiotics. For instance, Chaxalactins A–C, obtained from *Streptomyces* sp. C34, are active against Gram-positive bacteria—including *Staphylococcus aureus*, *Listeria monocytogenes*, and *Bacillus subtilis*—as well as showing weaker activity against Gram-negative bacteria such as *Escherichia coli* and *Vibrio parahaemolyticus* [17]. Three dithiolopyrrolone antibiotics—butanoyl-pyrrothine (BUP), senecioyl-pyrrothine (SEP), and tigloyl-pyrrothine (TIP)—were isolated from *Streptomyces algeriensis* SA 233 in the Sahara Desert [18]. These compounds not only inhibit Gram-positive bacteria (*Bacillus coagulans*, *Bacillus subtilis*, *Micrococcus luteus*) and Gram-negative bacteria (*Klebsiella pneumoniae*), but SEP and TIP also exhibit significant antifungal activity against *Saccharomyces cerevisiae*, *Mucor ramannianus*, and plant pathogenic fungi such as *Fusarium culmorum*, *Fusarium oxysporum* f. sp. *albedinis*, and *F. oxysporum* f. sp. *lini* [18]. However, continued exploration has frequently led to the re-isolation of known compounds [19,20]. The pressing need for novel compounds has driven the development of microbial resources from extreme environments, particularly desert-derived *Streptomyces*, as a promising solution.

Fortunately, researchers in our laboratory conducted a systematic investigation of microbial resources in Chinese deserts and identified strain HMX87^T^ through antibacterial activity assays, which exhibited outstanding antimicrobial properties. In this study, strain HMX87^T^ was isolated from the desert soil of the Tuha Basin and exhibited low 16S rRNA gene sequence similarity to known species. Through a polyphasic taxonomic approach incorporating phenotypic, physiological, biochemical, chemotaxonomic, and molecular phylogenetic analyses, the taxonomic status of the strain was determined. Furthermore, genomic analysis revealed that strain HMX87^T^ possesses a substantial number of genes associated with high-temperature tolerance, saline-alkaline resistance, and antimicrobial compound synthesis. These genetic features underscore its potential for producing bioactive metabolites and reflect its adaptive strategies to extreme environmental conditions, including high temperature, salinity, alkalinity, intense ultraviolet radiation, and oligotrophic nutrient availability.

## 2. Methods

### 2.1. Sampling and Cultivation and Phylogenetic Analysis of 16s rRNA

Strain HMX87^T^ was isolated from a desert soil sample collected in the Tuha Basin, China (43°23′53.45″ N, 91°48′56.84″ E, altitude 1183.49 m). Approximately 5 g of soil was suspended in 25 mL of sterile saline solution, followed by serial dilution using phosphate-buffered saline (PBS). A 100 μL aliquot of the suspension was spread onto Gauze’s No. 1 agar plates and incubated aerobically at 30 °C. Individual colonies with diverse morphologies were repeatedly streaked onto fresh Gauze’s No. 1 medium at 30 °C for purification. The resulting pure cultures were stored at −80 °C until further analysis. Strain HMX87^T^ was additionally cultivated on Gauze’s No. 1 agar for subsequent taxonomic characterization and genomic studies.

The two most closely related type strains of *Streptomyces* were acquired from the China General Microbiological Culture Collection Center (CGMCC; www.cgmcc.net, accessed on 8 September 2023) and the Deutsche Sammlung von Mikroorganismen und Zellkulturen (DSMZ; www.dsmz.de, accessed on 15 March 2024), and used for a series of comparative experiments with strain HMX87^T^. The 16S rRNA gene of strain HMX87^T^ was amplified and sequenced using primers 27F and 1492R, following previously described methods [21]. The resulting 16S rRNA sequence was compared against the EzBioCloud database (www.ezbiocloud.net, accessed on 21 August 2023) to identify the most similar sequences. Phylogenetic trees were reconstructed using MEGA version 7.0 [22].

### 2.2. Phenotypic and Biochemical Tests of Strain HMX87^T^

To characterize the phenotypic properties of strain HMX87^T^, it was cultivated on Gauze’s No. 1 agar medium, and its spore morphology was examined using scanning electron microscopy (SEM) after fixation with glutaraldehyde, dehydration through an ethanol series, critical-point drying, and gold coating, as described previously. A broad range of phenotypic traits were assessed using the Biolog GEN III MicroPlate system, in addition to conventional biochemical, physiological, and growth assays relevant to the genus *Streptomyces*. All phenotypic data were obtained from two or three independent replicates using commercial assay kits. Cellular fatty acids were extracted, methylated, and analyzed using the Microbial Identification System (MIDI) version 6.0 [23]. Polar lipids were separated by two-dimensional thin-layer chromatography (TLC) on silica gel plates. The first dimension was developed in chloroform/methanol/distilled water (65:25:4, *v*/*v*), and the second dimension was developed in chloroform/glacial acetic acid/methanol/distilled water (80:18:12:5, *v*/*v*). The plates were sprayed with specific detection reagents for the identification of different lipid classes: phosphomolybdate for total lipids, ninhydrin for aminolipids, molybdenum blue for phospholipids, and α-naphthol for glycolipids, according to the standard method of Minnikin et al. [24]. The composition of cell-wall diaminopimelic acid and whole-cell sugars was determined by TLC following established protocols [25]. Menaquinones were extracted from freeze-dried biomass and purified using the method outlined by Collins [26].

### 2.3. Genome Sequencing and Analysis

Genomic DNA was extracted using the Wizard Genomic DNA Purification Kit (Promega) following the manufacturer’s protocol. Genome sequencing of strain HMX87^T^ was performed using both PacBio RS II and Illumina HiSeq 2000 platforms at Majorbio Bio-Pharm Technology Co., Ltd. (Shanghai, China). A high-quality dataset was generated with approximately 100× coverage. Raw reads were preprocessed and filtered using the Illumina analysis pipeline [27]. De novo assembly was carried out using the Celera Assembler (version 8.0) [28], resulting in contigs that were further scaffolded into two final scaffolds. Gene prediction was performed using Glimmer [29], GeneMarkS [30], and Prodigal [31]. Predicted genes were functionally classified and assigned to metabolic pathways using BLASTP (version 2.17.0) [32] against the COG and KEGG databases. A circular genome map was generated with Circos [33]. Pan-genome analysis was performed using the OrthoVenn3 web platform (https://orthovenn3.bioinfotoolkits.net, accessed on 10 December 2024) with default parameters [34]. The average nucleotide identity (ANI) was computed using the ANI calculator available on the EzBioCloud platform [35]. Biosynthetic gene clusters (BGCs) for secondary metabolites were predicted with antiSMASH 5.0 [36].

### 2.4. Antibacterial Activity Assay

Antimicrobial activity was assessed using the standard agar diffusion assay. The detailed procedure was as follows: (1) Pathogenic indicator strains were inoculated into liquid LB medium and incubated with shaking at 37 °C until the logarithmic growth phase was reached (approximately 6 h). Bacterial suspensions were then adjusted to a concentration of 1 × 10^8^ to 1 × 10^9^ CFU/mL using the McFarland standard method. (2) Melted LB agar medium was cooled to 50 °C, inoculated with 2% (*v*/*v*) of the bacterial suspension, mixed thoroughly, and poured into Petri dishes. (3) Wells of 6 mm in diameter were made in the solid agar using a sterile borer, and 200 µL of the test sample was added into each well. (4) Plates were incubated at 37 °C for 8 h, after which the diameters of the inhibition zones were measured. All experiments were performed in three independent replicates.

## 3. Results

### 3.1. Phylogenetic Analysis Based on 16s rRNA Gene Sequences

**Figure 1:**
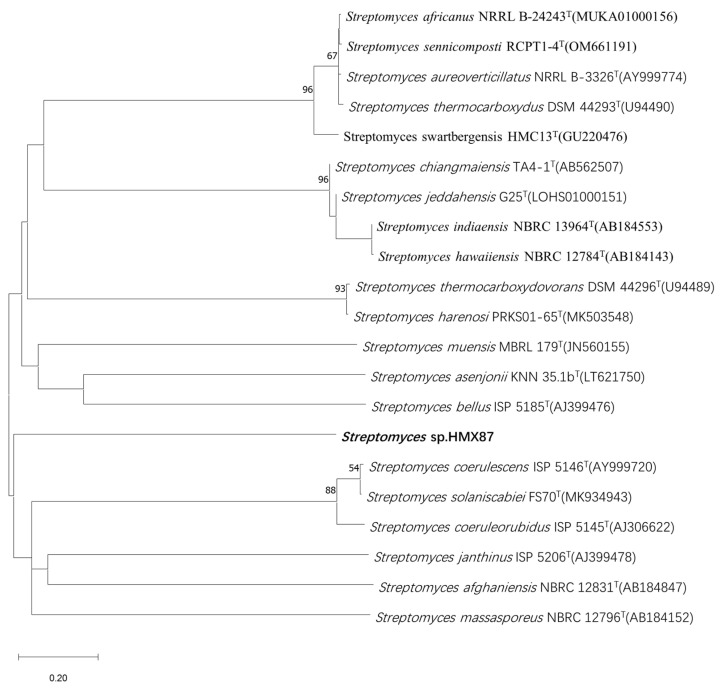
Phylogenetic tree constructed by neighbor-joining method between strain HMX87T and its closest type strain. Note: *Streptomyces bellus* ISP 5185 = CGMCC 4.1376^T^ and *S. coerulescens* ISP 5146 = DSM 40146^T^; these strains represent the same type strains preserved in different culture collections.

BLAST analysis of the complete 16S rRNA gene sequence (1527 bp) of strain HMX87^T^ against the EzBioCloud database revealed that it belongs to the genus *Streptomyces*. The highest sequence similarity was observed with *Streptomyces bellus* CGMCC 4.1376^T^ (98.5%), followed by *Streptomyces coerulescens* DSM 40146^T^ (98.43%). Both values are below the recommended 16S rRNA gene sequence similarity threshold of 98.65% for the delineation of novel *Streptomyces* species. A phylogenetic tree was reconstructed using the neighbor-joining method based on 16S rRNA gene sequences of 20 type strains exhibiting similarity greater than 97.79% with strain HMX87^T^. As shown in Figure 1, strain HMX87^T^ formed a distinct branch, separate from other closely related species, indicating its phylogenetic isolation and supporting its status as a potential novel species. Notably, *Streptomyces bellus* ISP 5185 corresponds to the same type strain as *S. bellus* CGMCC 4.1376^T^, and *S. coerulescens* ISP 5146 corresponds to the same type strain as *S. coerulescens* DSM 40146^T^, but preserved in different culture collections. The 16S rRNA gene sequence of strain HMX87^T^ has been deposited in GenBank under the accession number PQ182592.

### 3.2. Phenotypic and Chemotaxonomic Characterization

**Figure 2:**
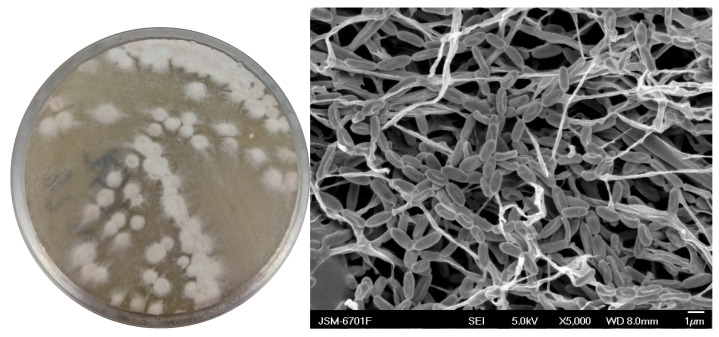
Colony morphology and SEM of strain HMX87.

Strain HMX87^T^ is a Gram-positive bacterium that exhibits robust growth on a variety of culture media, including Gauze’s No. 1, Tryptic Soy Broth (TSB), ISP-2, ISP-7, Minimal Salts (MS), Yeast Starch, and Brain Heart Infusion (BHI) agar. Optimal sporulation was observed on Gauze’s No. 1 agar, where the colonies appeared white in the initial stages and gradually turned light pink as they matured. The sporulating aerial mycelium exhibited a fluffy texture, forming extensive substrate mycelia along with abundant aerial hyphae. Scanning electron microscopy revealed that the spores are ellipsoidal in shape, with smooth surfaces, and are arranged in chains (Figure 2).

Strain HMX87^T^ exhibited a growth temperature range of 25–37 °C, with an optimum between 30 and 37 °C. It tolerated pH values from 5 to 12, with optimal growth observed at pH 7–8, and survived NaCl concentrations up to 8% (*w*/*v*). These physiological traits suggest that the strain is adapted to mesophilic saline-alkaline environments, consistent with the conditions of the Tuha Basin desert from which it was isolated. Carbon source utilization profiling using the Biolog GEN III MicroPlate identified the following utilizable substrates: Sugars (D-trehalose, D-cellobiose, gentiobiose, sucrose, D-turanose, stachyose, raffinose, α-d-lactose, melibiose, D-galactose, L-fructose, inositol, D-sorbitol, D-arabitol, glycerol, D-glucose-6-phosphate, D-fructose-6-phosphate, pectin, D-galacturonic acid, L-galactonic acid lactone, D-gluconic acid, and quinic acid); amino sugars and glycosides ( β-methyl-d-glucoside, D-salicin, N-acetyl-β-d-glucosamine, and N-acetyl-d-galactosamine); and organic acids (citric acid, methyl D-lactate, acetoacetic acid, propionic acid, and acetic acid). Nitrogen sources supporting growth included glycyl-l-proline, L-alanine, L-arginine, and L-serine, among others. In antibiotic susceptibility tests, the strain was sensitive to aztreonam but tolerant to nalidixic acid and lithium chloride. It demonstrated gelatin hydrolysis activity and exhibited strong antimicrobial properties against the tested pathogens. Detailed physiological and biochemical characteristics of strain HMX87 and its closely related type strains are summarized in Table 1.

**Figure 3:**
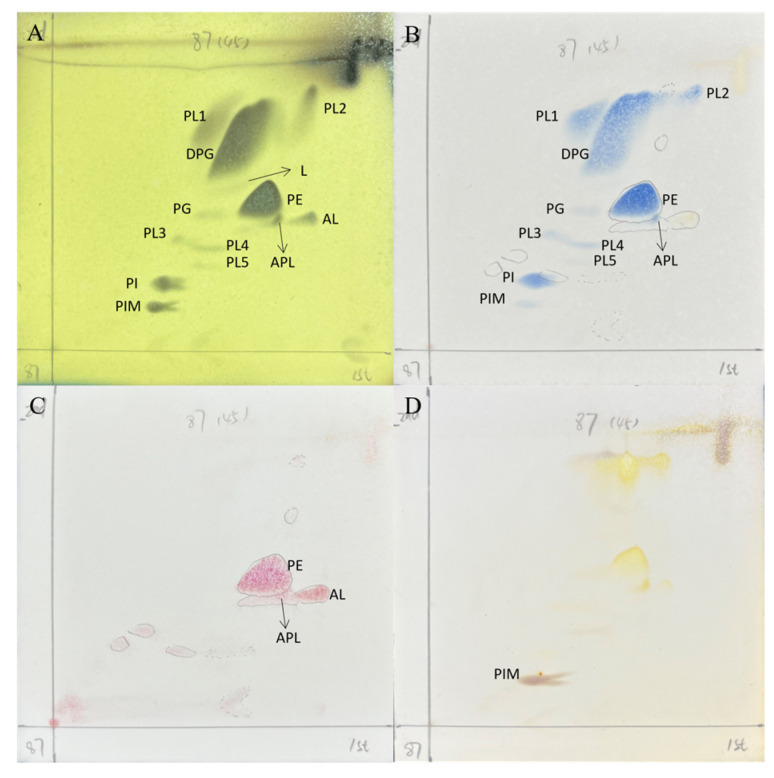
Thin-layer chromatography (TLC) analysis of polar lipids from strain HMX87^T^. Note: Chemical detection of polar lipids on the TLC plate was performed using the following specific spray reagents: (**A**) phosphomolybdate for total lipids; (**B**) molybdenum blue for phospholipids; (**C**) ninhydrin for aminolipids; (**D**) α-naphthol (1-naphthol) for glycolipids.

The cell wall of strain HMX87^T^ was found to contain LL-diaminopimelic acid (LL-DAP) as the predominant diamino acid, and its whole-cell hydrolysates primarily consisted of ribose and galactose. These characteristics are consistent with those typical of the genus *Streptomyces*. The major menaquinones identified were MK-9(H8) (66.3%), MK-9(H6) (13.57%), and MK-9(H4) (11.7%). The polar lipids detected included diphosphatidylglycerol (DPG), phosphatidylglycerol (PG), phosphatidylinositol (PI), phosphatidylethanolamine (PE), phosphatidylinositol mannosides (PIM), as well as unidentified phospholipids (PL), lipids (L), aminolipids (AL), and aminophospholipids (APL) (Figure 3). The predominant cellular fatty acids were iso-C_16:0_ (25.94%), anteiso-C_15:0_ (16.98%), and iso-C_15:0_ (7.01%). A detailed comparative analysis of the fatty acid composition of strain HMX87 and its closely related type strains is provided in Table 2.

### 3.3. Whole-Genome Sequencing of Strain HMX87^T^

**Figure 4:**
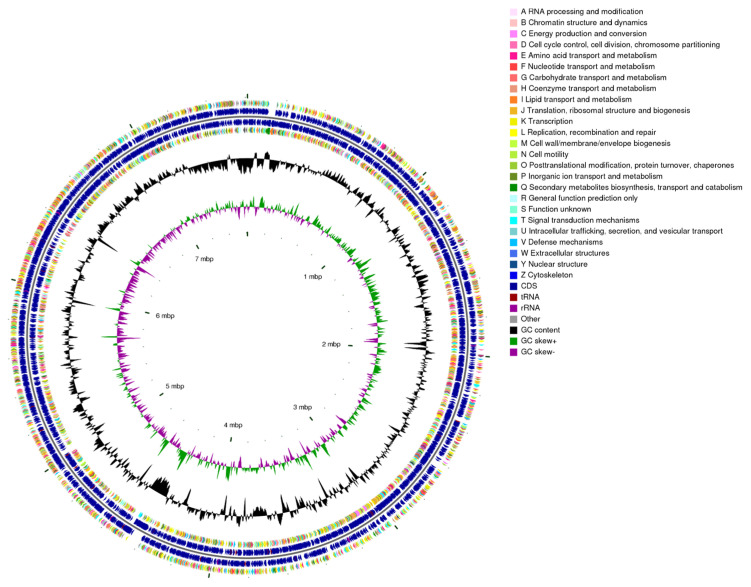
CGView of the genome of strain HMX87^T^.

Genome assembly revealed that the complete genome of strain HMX87^T^ consists of one circular chromosome and one plasmid, with a total size of 8,202,574 bp and a GC content of 72.2% (Figure 4). The genome contains 7228 protein-coding genes (CDSs), 18 rRNA genes, 63 tRNA genes, and 82 sRNA genes. To evaluate the taxonomic status of strain HMX87^T^, average nucleotide identity (ANI) and digital DNA–DNA hybridization (dDDH) values were calculated between HMX87^T^ and two closely related type strains of the genus *Streptomyces* (Table 3). Both ANI and dDDH values were well below the established species delineation thresholds of 95% and 70%, respectively. Based on a comprehensive polyphasic approach integrating phenotypic, physiological, biochemical, chemotaxonomic, and genomic characteristics, strain HMX87^T^ is proposed to represent a novel species of the genus *Streptomyces*, for which the name *Streptomyces hamibioticus* sp. nov. is assigned. The whole-genome sequence has been deposited in the NCBI GenBank database under the accession number PRJNA1206124.

### 3.4. Comparative Genomic Analysis of Three Streptomyces Species

**Figure 5:**
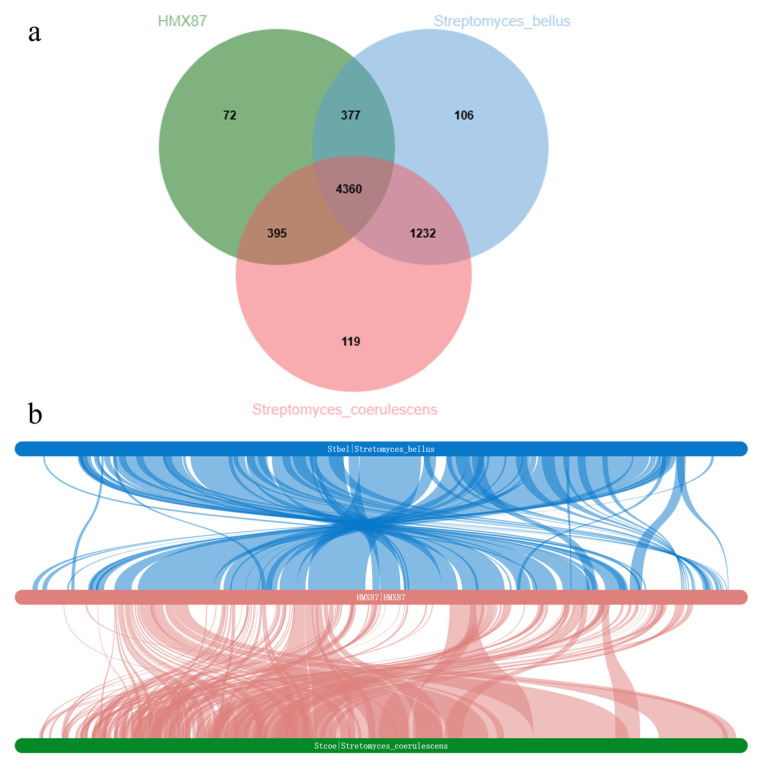
Pan-genomic analysis and genomic covariance analysis plots of three *Streptomyces* species. Note: (**a**) Venn diagram showing shared and unique genes among the three *Streptomyces* strains; (**b**) Sankey diagram illustrating gene flow and common gene relationships.

To investigate genomic differences and similarities between strain HMX87^T^ and its close relatives *Streptomyces bellus* CGMCC 4.1376^T^ and *Streptomyces coerulescens* DSM 40146^T^, a pan-genome analysis of their protein-coding genes was performed using OrthoVenn3. The results (Figure 5a) revealed that the three strains share 4360 common genes, accounting for the majority of the gene content in each strain, indicating a high degree of conservation in core metabolic and essential physiological functions among these closely related streptomycetes. Additionally, strain HMX87^T^ possesses 72 unique genes, which may be associated with its specific ecological adaptations and secondary metabolic capabilities. The Sankey diagram (Figure 5b) clearly indicates that strain 87 shares a greater number of genes with the *S. bellus* type strain (as shown by the thicker connecting lines), suggesting a closer evolutionary relationship between them than with *S. coerulescens*.

### 3.5. Genomic Features for Adaptation to Extreme Environments of Strain HMX87^T^

**Figure 6:**
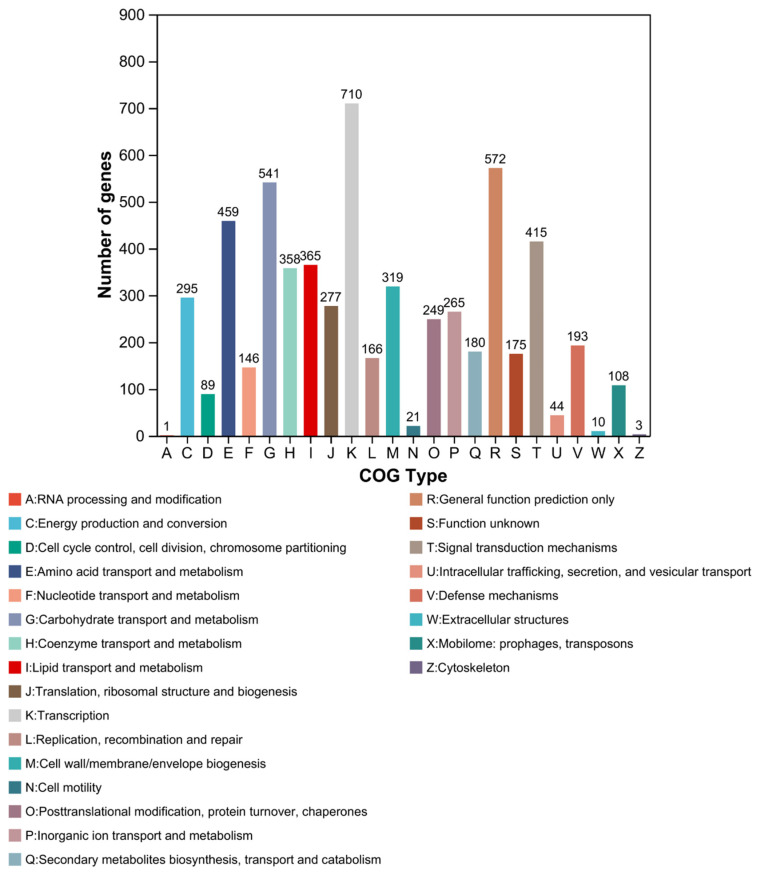
COG functional classification chart for strain HMX87^T^.

Among the 7228 predicted protein-coding genes of strain HMX87^T^, 5961 (82.47%) were functionally annotated into Clusters of Orthologous Groups (COG) categories (Figure 6). A wide functional diversity was observed, with particularly abundant roles in core metabolic processes: genes related to transcription (Category K) and translation, ribosomal structure, and biogenesis (Category J) were the most highly represented, encoding RNA polymerases, ribosomal proteins, and translation factors, indicating robust genetic expression and regulatory capacity. Significant enrichment was also detected in genes involved in carbohydrate transport and metabolism (Category G, 9.07%) and amino acid transport and metabolism (Category E, 7.7%), consistent with its broad carbon source utilization (e.g., D-cellobiose, D-trehalose) and nitrogen metabolic traits (e.g., L-alanine, L-serine).

Regarding environmental adaptation, genes associated with signal transduction mechanisms (Category T, 7.0%) were abundant, particularly two-component systems and stress-response regulators, suggests that strain HMX87^T^ may have an enhanced capacity to sense and respond to environmental fluctuations, such as changes in temperature and salinity. Genes for inorganic ion transport and metabolism (Category P, 4.4%) were also notably present, correlating with its salt tolerance (0–8% NaCl), and are predicted to encode Na^+^/K^+^ ion transport systems. In terms of secondary metabolic potential, 180 genes (3.0% of annotated genes) were assigned to secondary metabolite biosynthesis, transport, and catabolism (Category Q), including nonribosomal peptide synthetases (NRPS), polyketide synthases (PKS), and terpenoid biosynthesis gene clusters, reflecting a diverse capacity for synthesizing bioactive compounds. Defense mechanism genes (Category V, 3.2%), such as those encoding antibiotic resistance proteins and toxin efflux pumps, may contribute to adaptation under competitive environmental pressures including antibiotics and heavy metals. Other essential functions included cell wall/membrane/envelope biogenesis (Category M, 5.4%), consistent with its Gram-positive characteristics including a thick peptidoglycan layer, and genes for replication, recombination, and repair (Category L, 2.8%), indicative of genomic stability maintenance and DNA repair under environmental stress.

Overall, the COG functional profile of strain HMX87T reflects its metabolic adaptability to environments with fluctuating salinity and alkalinity, while its rich repertoire of secondary metabolic genes provides a genetic basis for the discovery of novel bioactive natural products. In addition, the abundance of genes related to signal transduction and stress response suggests that the strain can effectively perceive and respond to environmental changes, including moderate temperature variations.

### 3.6. Analysis of the Antibacterial Potential of Strain HMX87^T^

To explore the secondary metabolic potential of strain HMX87^T^, a systematic analysis of biosynthetic gene clusters (BGCs) was conducted on its complete genome sequence using antiSMASH 7.0. Prediction results (Table 4) revealed the presence of 30 BGCs in the genome, encompassing diverse types of compounds such as nonribosomal peptide synthetases (NRPS), polyketides (PKS), terpenes, nucleosides, and others. Among the detected BGCs, several exhibited high similarity to gene clusters responsible for known metabolites, including geosmin, albaflavenone, citrulassin D, desferrioxamine B/E, ectoine, flaviolin/1,3,6,8-tetrahydroxynaphthalene, and alkylresorcinol biosynthesis (100% similarity). Additionally, BGCs related to the production of hopene, isorenieratene, melanin, and streptamidine—classified as terpene, NI-siderophore, melanin, and RIPP types—showed 66% similarity. Furthermore, multiple BGCs with unknown functions or low similarity to known clusters were identified, including hybrid NRPS-PKS clusters that displayed only 16% similarity to cloipdibicyclene/azabicyclene-type compounds, suggesting their potential for novel metabolic activities. The widespread distribution of BGCs in the genome of strain HMX87^T^ includes both conserved clusters associated with known bioactive secondary metabolites and unique clusters that may be involved in the biosynthesis of new compounds, highlighting the strain’s rich genetic resources for further exploration and development of secondary metabolites.

## 4. Discussion

*Streptomyces* constitute a vital component of desert microbial communities, as evidenced by their high abundance, the continual discovery of novel taxa, and their widespread distribution across diverse desert environments [6,15,37]. The high GC content (72.2%) of strain HMX87^T^ may facilitate adaptation to high-temperature environments by enhancing DNA thermal stability [38], a trait consistent with evolutionary strategies commonly observed in extremophilic microorganisms. Enrichment of core metabolic genes—such as those involved in carbohydrate and amino acid metabolism—reflects the strain’s highly efficient utilization of limited nutrients in oligotrophic environments. Its preferential assimilation of specific substrates such as D-trehalose and L-serine is likely associated with the natural distribution of carbon and nitrogen sources within its native habitat. The optimal growth temperature of strain HMX87^T^ (30–37 °C) corresponds closely to the elevated temperatures characteristic of its desert origin. Genes encoding heat shock proteins and molecular chaperones (COG category O, 4.2%) likely contribute to its thermotolerance mechanisms. With regard to salt-alkali tolerance and osmotic regulation, genes related to signal transduction (category T, 7.0%) and inorganic ion transport (category P, 4.4%) may assist in maintaining cellular osmotic balance under high-salinity conditions through coordinated signaling and Na^+^/K^+^ ion transport. Additionally, the presence of an ectoine biosynthetic gene cluster (BGC24, 100% similarity) is likely strongly associated with its high salt-tolerance phenotype [39].

Moreover, desert-derived *Streptomyces* play a significant role in the discovery of bioactive natural products due to their extensive secondary metabolic capabilities. In strain HMX87^T^, COG annotation revealed that 3.0% of genes are associated with secondary metabolism (Category Q). antiSMASH analysis predicted numerous nonribosomal peptide synthetase (NRPS) and polyketide synthase (PKS) gene clusters, potentially encoding antimicrobial peptides or polyketides that may assist in suppressing competing microorganisms in nutrient-poor environments. Among these, BGC1 (NRPS, 16% similarity) may encode azabicyclene-like heterocyclic compounds, while BGC3 (LAP and thiopeptide, 10% similarity) could be involved in the synthesis of toxins or antibiotics. Additionally, high-similarity clusters such as BGC21 (desferrioxamine B/E, 100% similarity) are related to siderophore synthesis. The presence of siderophore-antibiotic complexes enhances antibacterial efficacy [40] and may aid the strain in competing for resources under iron-limited conditions. Defense mechanism genes (Category V, 3.2%) likely further improve its competitive advantage in extreme environments. Notably, low-similarity gene clusters such as BGC4 and BGC19 may represent novel metabolic pathways and the synthesis of previously uncharacterized compounds.

In summary, the findings of this study represent a significant addition to the microbial resource repository from extreme environments. They provide a reliable taxonomic foundation and high-quality biological materials for elucidating the ecological adaptation mechanisms of desert microorganisms to extreme stressors such as drought and high temperature, as well as for mining bioactive natural products with novel structures or functions. This work holds considerable significance for the field of microbial ecology and natural product discovery.

## 5. Conclusions

A *Streptomyces* strain was isolated from a soil sample collected in the Turpan-Hami Basin of Xinjiang, China. Genomic and phenotypic analyses revealed significant differences between strain HMX87^T^ and its closely related species, as evidenced by variations in Average Nucleotide Identity (ANI) and digital DNA-DNA Hybridization (dDDH) values, physiological characteristics, and polar lipid profiles, confirming its status as a novel species. Furthermore, the strain possesses various genes associated with environmental tolerance, and the characterization of its biosynthetic gene clusters (BGCs) for secondary metabolites demonstrates considerable potential for producing structurally diverse secondary metabolites with possible bioactivities.

Description of *Streptomyces hamibioticus* sp. nov.

*Streptomyces hamibioticus* (ha.mi.bi.o’ti.cus; N.L. masc. adj. hami-, referring to Hami City, China, where the strain was isolated; Gr. masc. adj. bioticus, pertaining to life; N.L. masc. adj. hamibioticus, indicating a microbe associated with life, possibly antibiotic-producing, isolated from Hami).

Cell are non-motile, aerobic, aerobic and strain Gram-positive. The spores are white in the early stages and slightly pink in the later stages when cultured in Gauze’s medium NO. 1. The spores have a velvety surface and form extensive basal mycelium and aerial mycelium. The growth temperature range of the strain HMX87^T^ is 25–37 °C (optimum range: 30–37 °C), with a pH tolerance of 5–12 (optimum: 7–8) and a salt (NaCl) tolerance of 0–8% (*w*/*v*). These physiological characteristics closely mirror the mesophilic, saline-alkaline environment of the Turpan-Hami Basin desert in Xinjiang, China, from which it was isolated. Metabolically, the strain efficiently utilizes a diverse range of carbon sources (including D-trehalose, D-cellobiose, and pectin) and nitrogen sources (such as L-alanine and L-serine). It demonstrates gelatin hydrolysis and exhibits sensitivity to aztreonam (positive result), while displaying tolerance to nalidixic acid and lithium chloride. Chemotaxonomically, the cell wall contains LL-diaminopimelic acid (LL-DAP). Whole-cell hydrolysates contain ribose and galactose as the diagnostic sugars. The predominant menaquinone is MK-9(H_8_) (66.3%). The major polar fatty acids are iso-C_16:0_ (25.94%) and -C_15:0_ (16.98%).

Strain HMX87^T^ has been deposited in the CCM and GDMCC culture collections under accession numbers CCM 9454^T^ and GDMCC 4.391^T^, respectively.

## Figures and Tables

**Table 1:** Physiological and biochemical characteristics of strain HMX87^T^ and its closely related type strains.

Test Characteristic	Strain HMX87^T^	*S. bellus* CGMCC 4.1376^T^	*S. coerulescens* DSM 40146^T^
Growth temperature	37 °C	25 °C	25 °C
pH tolerance	5–12	5–12	5–12
NaCl tolerance (*w*/*v*)	0–8	0–4	0–6
D-Trehalose	+	(+)	+
D-Cellobiose	+	(+)	(+)
Gentiobiose	+	(+)	+
Sucrose	+	(+)	+
D-Turanose	+	+	−
Stachyose	+	(+)	−
Raffinose	+	(+)	+
α-d-Lactose	+	(+)	−
Melibiose	+	+	+
D-Galactose	+	(+)	+
L-Fructose	+	(+)	−
Inositol	+	−	+
D-Sorbitol	+	(+)	+
D-Arabitol	(+)	+	(+)
Glycerol	+	+	+
D-Glucose-6-phosphate	+	+	−
D-Fructose-6-phosphate	+	+	−
Pectin	+	(+)	(+)
D-Galacturonic acid	+	+	−
L-Galactonic acid lactone	+	+	−
D-Gluconic acid	+	+	−
Quinic acid	+	+	−
β-Methyl-d-glucoside	+	(+)	−
D-Salicin	+	+	+
N-Acetyl-β-d-glucosamine	+	+	−
N-Acetyl-d-galactosamine	+	(+)	−
Citric acid	(+)	(+)	−
Methyl D-lactate	(+)	+	(+)
Bromo-succinic acid	−	+	−
Acetoacetic acid	+	+	−
Propionic acid	(+)	+	+
Acetic acid	(+)	(+)	(+)
Glycyl-l-proline	(+)	(+)	+
L-Alanine	+	(+)	−
L-Arginine	+	(+)	−
L-Serine	+	(+)	−
Aztreonam	+	+	+
Nalidixic acid	(+)	+	+
Lithium chloride	(+)	+	−
Gelatin	(+)	−	+
Tween 40	+	+	+
Antimicrobial activity	+	+	+

Note: +: Positive; −: Negative; (+): Borderline value.

**Table 2:** Cellular fatty acid composition of strain HMX87^T^ and its closest type strain.

Fatty Acid	Strain HMX87^T^	*S. bellus* CGMCC 4.1376^T^	*S. coerulescens* DSM 40146^T^
C_12:0_	0.11	0.11	0.50
C_13:0_	0.06	0.07	-
C_14:0_	0.79	0.82	1.46
C_15:0_	1.16	1.71	1.75
C_16:0_	10.3	6.41	11.16
C_17:0_	0.75	0.37	0.37
C_18:0_	0.27	0.10	-
iso-C_12:0_	0.22	0.1	0.18
iso-C_13:0_	0.24	0.33	0.48
iso-C_14:0_	3.90	4.35	4.11
iso-C_15:0_	7.01	15.69	14.7
iso-C_15:1_ F	-	0.06	-
iso-C_16:0_	25.94	17.37	18.95
iso-C_18:0_	1.17	1.89	1.43
iso-C_19:0_	-	0.19	-
anteiso-C_11:0_	0.12	-	-
anteiso-C_13:0_	0.52	0.24	0.43
anteiso-C_15:0_	16.98	17.93	15.47
anteiso-C_15:1_ A	0.20	-	-
anteiso-C_16:0_	0.16	0.11	-
anteiso-C_19:0_	0.04	-	-
iso-C_16:0_ 3OH	0.12	-	-
iso-C_16:1_ H	3.45	4.05	3.12
Sum In Feature 3	2.27	3.92	5.49
Sum In Feature 5	2.16	0.14	-
Sum In Feature 7	0.06	0.05	-
Sum In Feature 8	0.06	0.18	-
Sum In Feature 9	1.26	5.97	4.16
iso-C_17:0_	3.25	2.90	2.92
anteiso-C_17:0_	12.37	5.49	5.41
cyclo-C_17:0_	0.95	2.25	2.51
anteiso-C_17:1_ Ꞷ9c	2.29	4.90	3.56
Summed Feature 7	0.06	-	-
Summed Feature 8	0.06	0.23	-
Summed Feature 9	1.26	5.97	4.16

Note: Fatty acids are indicated using standard abbreviations; iso- and anteiso- denote branched chains, 3OH indicates hydroxylation, and double-bond positions denote unsaturation. “Sum in Feature X” represents the total percentage of fatty acids included in Feature X as defined by the MIDI system (v6.0). “Summed Feature 7, 8, 9” correspond to co-eluting fatty acids that cannot be resolved individually by GC. “-”: not detected. Values in the table represent percentages.

**Table 3:** Comparative analysis of 16S rRNA similarity, ANI, and digital dDDH values between Strain HMX87^T^ and closely related type strains.

Strain 1	Strain 2	16S rRNA Similarity (%)	ANI (%)	dDDH (%)
HMX87^T^	*S. bellus* CGMCC 4.1376^T^	98.5	84.4	32.1
	*S. coerulescen* DSM 40146^T^	98.43	83.28	22.9

**Table 4:** BGCs of secondary metabolites identified in strain HMX87^T^.

BGC	Type	Similar Known Cluster	Similarity
1	NRPS	clipibicyclene/azabicyclene B/azabicyclene C/azabicyclene D	16%
2	NRPS	zorbamycin	8%
3	LAP, thiopeptide	toxoflavin/fervenulin	10%
4	crocagin, T1PKS	-	-
5	terpene	-	-
6	RiPP-like	informatipeptin	57%
7	NRPS-like, betalactone	longicatenamide B/longicatenamide C/longicatenamide A/longicatenamide D	15%
8	terpene	hopene	92%
9	T1PKS, hydrogen-cyanide	aborycin	28%
10	CDPS	nogalamycin	40%
11	terpene, NI-siderophore	isorenieratene	85%
12	terpene	geosmin	100%
13	RiPP-like	-	-
14	T1PKS	auroramycin	8%
15	NI-siderophore	kinamycin	16%
16	terpene	albaflavenone	100%
17	NRP-metallophore, NRPS, lassopeptide	citrulassin D	100%
18	redox-cofactor	berninamycin K/berninamycin J/berninamycin A/berninamycin B	27%
19	phosphonate	-	-
20	lanthipeptide-class-i	-	-
21	NI-siderophore	desferrioxamin B/desferrioxamine E	100%
22	melanin	melanin	60%
23	NRPS	-	-
24	ectoine	ectoine	100%
25	T3PKS	flaviolin/1,3,6,8-tetrahydroxynaphthalene	100%
26	T1PKS	enduracidin	10%
27	RiPP-like	streptamidine	66%
28	melanin	melanin	57%
29	indole	5-isoprenylindole-3-carboxylate β-d-glycosyl ester	23%
30	T3PKS	alkylresorcinol	100%

## Data Availability

The original contributions presented in this study are included in the article. Further inquiries can be directed to the corresponding author.
